# Advances and prospects for treatment strategies of drug-resistant tuberculosis: a review

**DOI:** 10.3205/dgkh000562

**Published:** 2025-06-26

**Authors:** Maciej Michalik, Tomasz Lorenc, Krzysztof Marcinkowski, Mateusz Muras, Natalia Mikszta, Jakub Mikszta, Karolina Kantor, Julia Marcinkowska

**Affiliations:** 1 Warsaw Southern Hospital, Warsaw, Poland; 2 Ludwik Rydygier Specialist Hospital, Cracow, Poland; 3 Provincial Specialist Hospital Nr 2, Jastrzebie-Zdroj, Poland; 4 Provincial Specialist Hospital Nr 3, Rybnik, Poland; 5 Faculty of Medical Sciences in Katowice, Medical University of Silesia, Katowice, Poland

**Keywords:** drug-resistant tuberculosis (DR-TB), multidrug-resistant tuberculosis (MDR-TB), extensively drug-resistant tuberculosis (XDR-TB), BPaLM, WHO

## Abstract

Drug-resistant tuberculosis (DR-TB) poses a significant global health threat, particularly in low- and middle-income countries with limited access to quality healthcare. By 2023, 10% of global tuberculosis cases were classified as drug-resistant, with multidrug-resistant (MDR-TB) and extensively drug-resistant tuberculosis (XDR-TB) showing increasing prevalence.

The treatment of DR-TB has been complicated by long regimens, severe side effects and high overall costs, which contribute to non-adherence and treatment failures. Novel pharmacological agents including bedaquiline, linezolid, meropenem and more, have shown promise in improving treatment outcomes, shortening therapy duration, and enhancing patient compliance. These drugs have demonstrated effectiveness in both MDR-TB and XDR-TB cases, particularly when used in combination therapies as BPaLM (the combination of bedaquiline, pretomanid, linezolid and moxifloxacin).

However, challenges remain, including limited access to drugs, diagnostic tools, and healthcare infrastructure, particularly in high-burden regions. Although regimens incorporating these agents offer improved treatment success rates, they require careful monitoring due to potential side effects and the risk of resistance. Future research should focus on refining these regimens, optimizing drug use for resource-limited settings, and addressing logistical and economic barriers to ensure more effective and accessible treatment. The ultimate goal is to reduce the global burden of DR-TB and improve outcomes for affected populations.

## Abstract (de)

Die arzneimittelresistente Tuberkulose (DR-TB) stellt eine erhebliche globale Gesundheitsbedrohung dar, insbesondere in Ländern mit niedrigem und mittlerem Einkommen, die begrenzten Zugang zu hochwertiger Gesundheitsversorgung haben. Bis 2023 wurden 10% der weltweiten Tuberkulosefälle als arzneimittelresistent eingestuft, wobei die Prävalenz von multiresistenter (MDR-TB) und extensiv arzneimittelresistenter Tuberkulose (XDR-TB) zunimmt.

Die Behandlung der DR-TB wird durch langwierige Therapieschemata, schwere Nebenwirkungen und hohe Gesamtkosten erschwert, was zu mangelnder Therapieadhärenz und Behandlungsversagen führt. Neuartige pharmakologische Wirkstoffe wie Bedaquilin, Linezolid, Meropenem und andere haben vielversprechende Ergebnisse hinsichtlich der Verbesserung der Therapieergebnisse, der Verkürzung der Therapiedauer und der Erhöhung der Patientenzufriedenheit gezeigt. Insbesondere in Form von Kombinationstherapien wie BPaLM (die Kombination von Bedaquilin, Pretomanid, Linezolid und moxifloxacin) haben sich diese Medikamente bei MDR-TB- und XDR-TB-Fällen als wirksam erwiesen.

Dennoch bestehen weiterhin Herausforderungen durch eingeschränkten Zugang zu Medikamenten, Diagnosetools und medizinischer Infrastruktur, insbesondere in Regionen mit hoher Krankheitslast. Obwohl Therapieschemata, die diese Wirkstoffe integrieren, höhere Erfolgsraten bieten, erfordern sie eine sorgfältige Überwachung aufgrund potenzieller Nebenwirkungen und des Risikos der Resistenzentwicklung. Zukünftige Forschung sollte darauf abzielen, die Situation zu optimieren, den Einsatz von Medikamenten in ressourcenarmen Umgebungen zu verbessern und logistische sowie wirtschaftliche Barrieren anzugehen, um effektivere und zugänglichere Behandlungen sicherzustellen. Das übergeordnete Ziel bleibt die Reduktion der globalen DR-TB-Belastung und die Verbesserung der Behandlungsergebnisse für die betroffenen Bevölkerungsgruppen.

## Introduction

Drug-susceptible tuberculosis (DS-TB) refers to tuberculosis cases that are responsive to standard first-line treatment, which typically involves a six-month regimen of isoniazid, rifampicin, ethambutol, and pyrazinamide [1], [2], [3]. In contrast, drug-resistant tuberculosis (DR-TB) includes cases of tuberculosis that exhibit resistance to one or more of these first-line drugs [1], [2], [4]. In 2023, approximately 10% of global tuberculosis cases were classified as drug-resistant, with higher prevalence rates observed in low- and middle-income countries, where access to quality healthcare remains a significant obstacle [1], [5],[6] . 

By 2023, one in every ten newly diagnosed tuberculosis cases worldwide were found to be resistant to at least one drug, with an increasing proportion categorized as multidrug-resistant tuberculosis (MDR-TB) and extensively drug-resistant tuberculosis (XDR-TB). This growing trend presents considerable challenges in the global effort to treat tuberculosis [1], [6], [7]. MDR-TB is particularly prevalent in low- and middle-income countries, including India, China, and Russia, where healthcare access is constrained, and treatment interruptions are more common [3]. 

Drugs recommended for use in standard and extended MDR-TB treatment regimens are organized according to their preferability:

- Group A: levofloxacin or moxifloxacin, bedaquiline and linezolid;
- Group B: clofazimine, and cycloserine or terizidone;
- Group C: ethambutol, delamanid, pyrazinamide, imipenem-cilastatin or meropenem, amikacin (or streptomycin), ethionamide or prothionamide, and p-aminosalicylic acid (Table 1 (Tab. 1)).

Currently, the WHO recommends that clinicians prioritize shorter, safer, and more effective treatment regimens [7]. 

## Risk factors for MDR-TB

Regions such as Eastern Europe, Southeast Asia, and sub-Saharan Africa are heavily burdened by DR-TB. In these areas, challenges such as limited diagnostic resources, issues with treatment adherence, and constrained healthcare systems contribute significantly to the spread and persistence of MDR-TB [4], [5]. Additionally, HIV co-infection and malnutrition are recognized risk factors for the development of DR-TB. Both conditions undermine immune function and treatment efficacy, thus increasing the likelihood of drug resistance development [7], [8], [9], [10], [11]. 

Diabetes mellitus (DM) is another key factor for the incidence of DR-TB. Individuals with DM are more vulnerable to MDR-TB due to compromised immune responses, leading to poorer treatment outcomes and an increased risk of drug resistance. Evidence indicate that these factors correspond with an elevated risk of both MDR-TB and treatment failure in aforementioned populations [12], [13], [14], [15]. 

Previous TB infections or incomplete treatment, whether resulting from patient non-compliance or treatment interruptions, are well-documented risk factors for the emergence of drug-resistant tuberculosis strains [16], [17], [18], [19], [20]. Furthermore, lifestyle factors such as smoking and alcohol use can impair immune function, further increasing the risk of developing DR-TB [16], [21], [22]. 

## Challenges in the treatment of DR-TB

The management of DR-TB, particularly MDR-TB and XDR-TB, presents substantial obstacles. MDR-TB is characterized by resistance to at least isoniazid and rifampicin, the two most effective first-line drugs. XDR-TB is even more concerning as it is resistant not only to isoniazid and rifampicin, but also to at least one fluoroquinolone and either bedaquiline or linezolid, two crucial second-line agents [1], [2]. 

The presence of XDR-TB severely limits treatment options, necessitating lengthy regimens with second-line drugs, many of which are associated with rare, yet at times severe side effects, some of which include gastrointestinal disturbances, liver toxicity, hearing loss, and renal impairment [23], [24], [25]. While treatment for drug-susceptible tuberculosis (DS-TB) typically spans 6 months, therapy for MDR-TB and XDR-TB can last from 6 months under novel shorter regimens to as long as 18 months in traditional regimens, due to the complexity and extended duration of second-line drug use [2], [3]. 

The high cost of these medications, along with their side effects and the problematic nature of the treatment regimens, significantly contribute to non-adherence. Furthermore, diminished compliance, often resulting from treatment fatigue and inadequate patient education, compounds treatment failures and heightens the risk of further resistance, given reports of WHO’s XDR-TB cases underestimation [4], [26], [27], [28]. Socioeconomic factors such as poverty, malnutrition, and weak healthcare infrastructures, alongside poor education, exacerbate the difficulties in ensuring adherence, particularly in regions with limited access to healthcare services [1], [27], [28]. A lack of sufficient patient support, especially among vulnerable populations, further deteriorates treatment outcomes, fueling the loop that contributes to the increaced risk of complications [1], [28]. 

Additionally, insufficient diagnostic capabilities, including the slow rollout of rapid diagnostic tests, lead to delays in treatment initiation, often allowing resistant strains to spread undetected [1], [29]. Despite advancements in treatment strategies, DR-TB continues to pose a significant global health threat, with the global treatment success rate still hovering around 44% – far below the target of 75% [1], [30]. This underscores the critical need for personalized treatment regimens that consider drug resistance patterns, comorbid conditions, and patient adherence. The continued suboptimal treatment success rate highlights the necessity for ongoing research and the development of innovative therapies and carefully tailored regimens to improve outcomes for DR-TB patients worldwide [1], [12], [24], [26], [31]. 

## Bedaquiline as a core component of BPaLM strategies and novel regimen patterns

Bedaquiline is a member of the diarylquinolone class, a new generation of compounds that effectively target *M. tuberculosis* by inhibiting the proton pump of the mycobacterial ATP-synthase. Its action is primarily channelled through binding to the subunit encoded by the atpE gene (subunit c) [32], [33]. As a novel agent, bedaquiline was introduced to the WHO-endorsed first-line treatment regimen for DR-TB [34], following its approval by the FDA for the treatment of *M. tuberculosis* after a decade of use [35]. Its rapid incorporation into treatment protocols was supported by numerous reports of culture conversion and high treatment success rates in patients receiving bedaquiline-containing regimens for DR-TB, including the salvage therapies for patients with previous treatment failures, unfavorable resistance profiles, and/or toxicity history [23], [36], [37]. 

The side effects of bedaquiline, although relatively common, primarily include nausea, peripheral neuropathy, kidney damage, and otovestibular toxicity [37], [38]. Reports of elevated incidence of QT interval prolongation call for caution regarding combination of bedaquiline with other drugs known to prolong the QT interval – including clofazimine used in some DR-TB regimens [39]. In some cases, more than 20% of patients required complete discontinuation of the drug due to adverse reactions [24]. 

The WHO’s consolidated guidelines from 2022 recommend a 6-month treatment regimen comprising bedaquiline, pretomanid, linezolid (600 mg) as a core scheme (BPaL) and moxifloxacin (BPaLM), as an alternative to the longer 9–20 month regimens for MDR-TB. In cases of documented fluoroquinolone resistance, the same regimen is recommended without moxifloxacin (the BPaL regimen) [7]. The longer duration of treatment is associated with increased non-compliance and patient drop-off, which suggests that shorter, effective, and safe regimens could significantly enhance the overall success rate of DR-TB treatment [40]. 

The BPaLM regimen has demonstrated superior efficacy and a more favorable safety profile compared to standard treatment regimens, particularly in patients with rifampin-resistant tuberculosis (RR-TB) [41]. Additionally, a controlled trial of a 24-week, all-oral regimen combining bedaquiline, pretomanid, and linezolid for DR-TB, specifically RR-TB, showed that BPaLM not only proved more effective but also resulted in fewer adverse events compared to the standard 9–20 month regimen [42]. Furthermore, reports indicate that this therapeutic approach may be more cost-effective long term [43], [44]. 

Practical challenges to the widespread adoption of bedaquiline-based regimens include limited access to the drug in certain regions and insufficient availability of drug susceptibility testing tools [45]. Moreover, the risk of acquired resistance to bedaquiline – and subsequently to regimens containing it – arises from its slow early bactericidal activity (EBA) [46] and its exceptionally long half-life [47], both of which could potentially contribute to resistance development due to accumulation over time. Ongoing clinical trials, coupled with real-world applications, will be crucial in further refining these regimens to optimize both their safety and efficacy. 

Regarding the quinolone component of the BPaLM regimen, while moxifloxacin is used, levofloxacin is also considered a viable alternative, yielding similar efficacy in treating DR-TB. From a safety perspective, levofloxacin is preferred over moxifloxacin due to the latter’s higher risk of cardiotoxicity, despite levofloxacin’s potential association with musculoskeletal adverse events in pediatric populations [48], [49]. Further research is needed to evaluate whether the substitution of quinolones in the BPaLM strategy proves to be both safe and effective. 

## Clofazimine Integration into BPaL-based and standard DR-TB treatment strategies

Clofazimine, a riminophenazine, is used alongside other agents such as bedaquiline, linezolid, quinolones (levofloxacin/moxifloxacin), and pretomanid in the treatment of MDR-TB and XDR-TB, providing a critical approach to resistant TB strains [50], [51]. Prior to the WHO’s 2022 guidelines [7], one study suggested that MDR-TB patients treated with clofazimine achieved comparable results to standard therapy, with the added benefit of a faster sputum conversion rate. The regimen in this study involved substituting ethambutol with clofazimine, reducing the duration of complete therapy from 18 to 12 months [52], [53]. 

The incorporation of clofazimine into MDR-TB and XDR-TB treatment regimens has been shown to improve treatment outcomes, leading to higher treatment completion and cure rates [51], [54], [55]. The most common adverse effect of clofazimine is skin discoloration [55], [56], although dosage adjustments can mitigate the frequency of these incidents [4]. Other adverse events, such as QT interval prolongation, gastrointestinal discomfort, liver damage, have been reported, although larger-scale trials are needed to better understand the prevalence of these effects [39], [50], [52], [54], [56], [57]. 

Currently, clofazimine is included as a variable in BPaL therapies, forming the bedaquiline, pretomanid, linezolid, clofazimine (BPaLC) regimen. Similarly to BPaLM, the BPaLC regimen, has a shortened duration of 24 weeks, which has proven to be more compliance-friendly and has outperformed the traditional 9–20 month regimen in terms of quality of life and cost-effectiveness [7], [58]. While the overall efficacy of clofazimine is difficult to assess due to its role as a supplement to MDR-TB and XDR-TB treatment regimens, evidence suggests that its addition holds promise in managing *M. tuberculosis* strains resistant to conventional therapies, whether as part of the BPaLC regimen or in older standardized regimens when the former is not available [7]. 

## Novel oxazolidinones and linezolid as a cornerstone in modern DR-TB treatment

According to current WHO guidelines, the treatment of DR-TB, including MDR-TB cases, requires a tailored approach, with the recommended use of shortened regimens such as BPaL strategies [7]. Linezolid, originally developed to treat Gram-positive bacterial infections, has been repurposed as a key component in modern regimens targeting *M. tuberculosis*. Its mechanism of action involves inhibiting bacterial protein synthesis by binding to the 23S ribosomal RNA of the 50S subunit, thereby disrupting bacterial growth [59]. However, the impact of linezolid on ribosomal RNA is not limited to bacteria; it also inhibits mitochondrial protein synthesis in human cells, which can lead to adverse effects associated with mitochondrial dysfunction [60]. These include and result in lactic acidosis, myelosuppression, and peripheral neuropathy. The severity of these side effects often necessitates discontinuation of the drug, resulting in treatment interruptions that reduce the overall efficacy of linezolid [59], [61]. 

Despite its proven effectiveness, the adverse events associated with linezolid have driven the search for newer, safer alternatives with similar mechanisms of action and efficacy [59]. Novel oxazolidinones, such as sutezolid, tedizolid, delpazolid, and TBI-223, show promise as potential replacements for linezolid in the treatment of linezolid-resistant strains of *M. tuberculosis* [62]. Although most of these new oxazolidinones have yet to complete clinical trials, initial studies on sutezolid have demonstrated adequate bactericidal activity in both blood and sputum, along with a more favorable safety profile [59], [63]. A double-blind, randomized controlled trial on orally administered sutezolid also indicated its safety in healthy volunteers [64]. While these findings are promising, they underscore the urgent need for further *in vitro*, *in vivo* and, importantly, subsequent clinical trials to assess the safety, efficacy, and optimal dosing of these drugs, as the path to practical implementation is a time-consuming process. 

## Meropenem and clavulanate use in DR-TB: prospects of new alternative carbapenem drugs

Meropenem, a carbapenem antibiotic, in combination with clavulanate, a β-lactamase inhibitor, has emerged as a promising option for treating DR-TB, including XDR-TB strains. This combination acts by inhibiting cell wall synthesis in *M. tuberculosis*, effectively targeting resistant strains that are otherwise unresponsive to first- and second-line anti-TB drugs [65], [66], [67], [68].

Studies demonstrate that meropenem-clavulanate exhibits strong bactericidal activity against both replicating and non-replicating *M. tuberculosis*, addressing a critical challenge in treating persistent bacteria [69]. The combination has shown efficacy, achieving reductions in bacterial load and enhancing sputum conversion rates [70]. The trial evaluating EBA reported that meropenem-clavulanate, with or without rifampin, achieved measurable reductions in *M. tuberculosis* burden within the first two weeks of treatment [65]. Additional *in vitro* studies highlight the combination’s potent activity against a wide range of DR-TB isolates, including strains with minimal susceptibility to other carbapenems [71]. Despite its efficacy, the regimen has limitations, including the need for intravenous administration, which may hinder its use in resource-limited settings. Nevertheless, ongoing research is exploring the development of oral carbapenems and alternative β-lactamase inhibitors to improve accessibility and practicality [69]. Furthermore, the potential limitation in establishing meropenem-clavulanate therapy would be lack of availability of sole clavulanate product other than one fused with amoxicillin which deems it impractical to prescribe it for TB unless one wants to use the combination with amoxicillin. Overall efficacy without potential addition of amoxicillin and poor overall tolerance of amoxicillin manifested with gastrointestinal adverse effects appear to be main obstacles in establishing this method as preferable. [72]

Meropenem-clavulanate unveils as a crucial addition to the arsenal against DR-TB, particularly for patients with limited treatment options. Its ability to target resistant *M. tuberculosis* and support rapid bacterial clearance offers hope for improving treatment outcomes in XDR-TB and MDR-TB cases [68]. Future studies focusing on optimizing dosing, exploring oral formulations, and integrating this combination into standardized regimens will further refine its role in TB management.

Other, novel carbapenem antibiotics (ertapenem, faropenem and tebipenem) were tested *in vitro* for their efficacy against MDR-TB and XDR-TB, that also included meropenem-resistant strains. The drugs’ efficacy was deemed dose-dependent with tebipenem as the most efficient one [73]. Further trials including possible future ones in the clinical setup are necessary to evaluate optimal dosage, efficacy and safety.

## Delamanid as a valid support in MDR-TB and XDR-TB treatment strategies

Delamanid is a novel drug developed for the treatment of MDR-TB and XDR-TB, specifically targeting strains resistant to first-line TB drugs [74]. It works by inhibiting the synthesis of methoxy- and keto-mycolic acids, which are essential components of the *M. tuberculosis* cell wall, thereby effectively targeting resistant strains of *M. tuberculosis* [74], [75]. 

Clinical studies have shown that delamanid significantly improves treatment outcomes, particularly when combined with bedaquiline [76]. This combination reduces the risk of treatment failure [36], [77], offering the potential for a therapeutic approach that is both safe and of shorter duration [76]. A minimum of six months of treatment with delamanid, when combined with an optimized background regimen, is associated with improved treatment outcomes and reduced mortality rates in DR-TB and XDR-TB cases [78], [79], [80]. In terms of efficacy, delamanid, when used in the appropriate setup, yields promising results [81]. However, side effects such as QT interval prolongation and liver enzyme abnormalities, while relatively rare even in salvage therapies involving delamanid and bedaquiline, necessitate careful monitoring during treatment [36], [74], [82]. There are also reports indicating that adverse effect rates may be higher when delamanid is used in combination therapy compared to monotherapy [83]. A range of trial results confirm that incorporating delamanid into MDR-TB treatment regimens enhances sputum conversion rates [74], [75], [84], [85], reduces mortality rates, and significantly improves overall treatment outcomes [36], [74], [80], [86]. 

Delamanid has become a critical drug in treating drug-resistant TB, especially for patients with strains resistant to most first- and second-line medications. As part of combination regimens, delamanid demonstrates high efficacy, particularly when used as the addition to other second-line agents such as bedaquiline, linezolid, clofazimine [74]. However, potential challenges to establishing delamanid as a standard component of DR-TB and XDR-TB therapies include reports of resistance mutations associated with delamanid-bedaquiline combinations [87], even in countries where these drugs are not yet widely used. These mutations could pose a threat to vulnerable populations in such regions. Additionally, the lack of established minimal inhibitory concentration (MIC) guidelines for determining resistance presents a significant concern and may necessitate further clinical trials to address these issues [88], [89]. 

## Conclusions

The battle against DR-TB continues to represent a significant global health challenge, due to the complexities associated with prolonged treatment durations, potential side effects and socio-economic factors that impact patient outcomes. Recent advancements in pharmacological regimens, which either incorporate novel chemotherapeutics or reintroduce previously used drugs, hold promise for improving treatment effectiveness. Agents such as bedaquiline, oxazolidinones, clofazimine, delamanid, and carbapenems have laid the groundwork for more efficient, shorter treatment protocols. These regimens have demonstrated potential in shortening therapy durations, enhancing bactericidal activity, improving treatment adherence, and increasing patient compliance. Nevertheless, the widespread adoption of these treatments is impeded by inadequate healthcare infrastructure, particularly in low- and middle-income countries where DR-TB prevalence remains highest. To overcome these barriers, a robust healthcare support system, along with a commitment to optimizing these regimens for both efficacy and accessibility, is essential. Ongoing research should focus on refining these treatment strategies to ensure their effectiveness and adaptability in resource-constrained environments, with the ultimate goal of reducing the global burden of drug-resistant tuberculosis and improving outcomes for affected populations worldwide.

## Notes

### Competing interests

The authors declare that they have no competing interests.

### Funding

None. 

### ORCIDs

- Michalik M: https://orcid.org/0009-0009-7799-7252
- Lorenc T: https://orcid.org/0009-0008-9902-469X
- Marcinkowski K: https://orcid.org/0009-0002-5759-7285
- Muras M: https://orcid.org/0009-0005-1392-3773
- Mikszta N: https://orcid.org/0009-0003-8444-7650
- Mikszta j: https://orcid.org/0009-0000-4194-9915
- Kantor K: https://orcid.org/0009-0005-0484-2883
- Marcinkowska J: https://orcid.org/0009-0005-7006-4303

## Figures and Tables

**Table 1:**
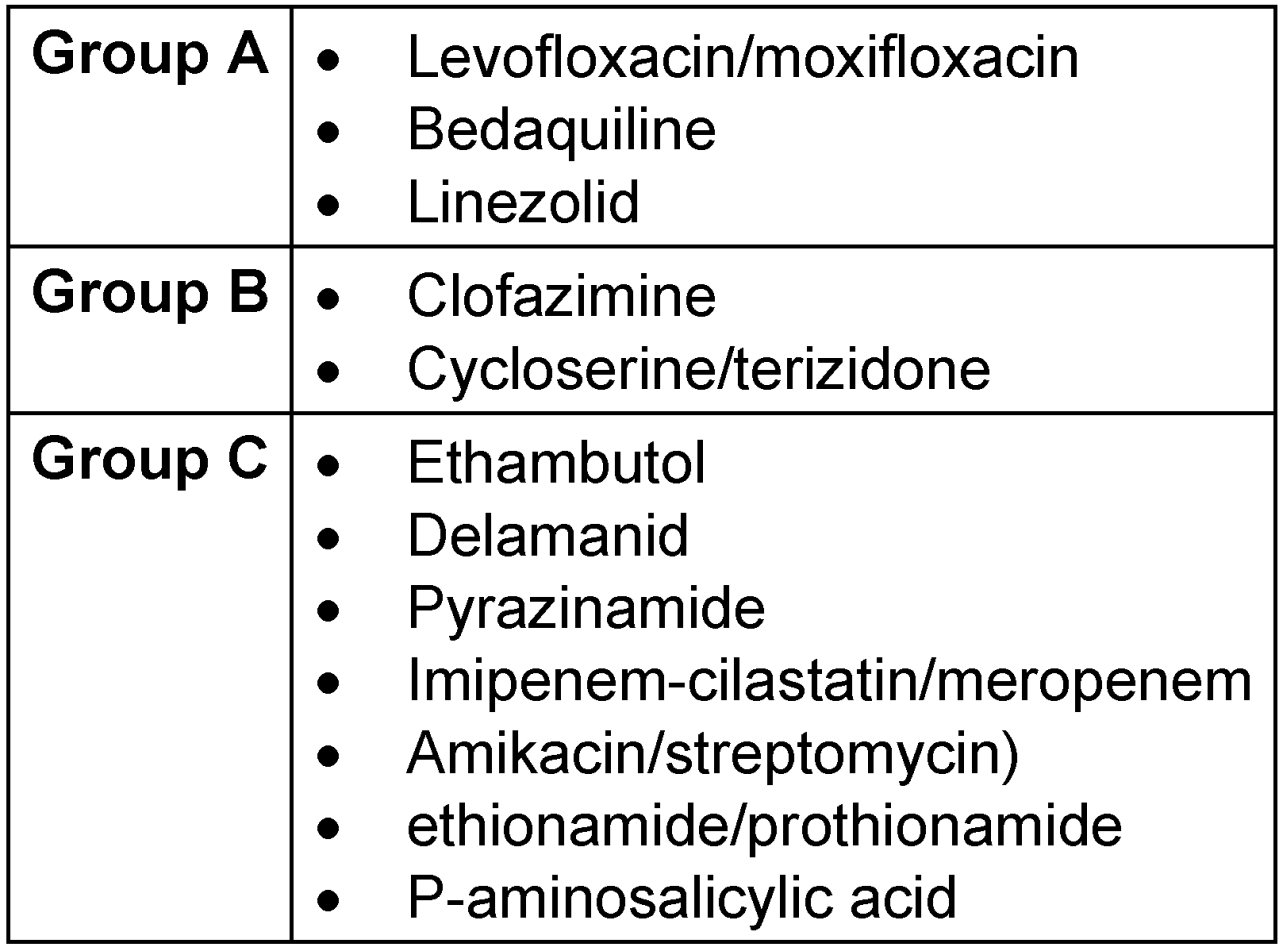
Based on WHO 2022, medication used in standard; longer MDR-TB (the slash mark indicates variables in choice)

## References

[1] World Health Organization (2024). Global Tuberculosis Report 2024.

[2] Paton NI, Cousins C, Suresh C, Burhan E, Chew KL, Dalay VB, Lu Q, Kusmiati T, Balanag VM, Lee SL, Ruslami R, Pokharkar Y, Djaharuddin I, Sugiri JJR, Veto RS, Sekaggya-Wiltshire C, Avihingsanon A, Sarin R, Papineni P, Nunn AJ, Crook AM, TRUNCATE-TB Trial Team (2023). Treatment Strategy for Rifampin-Susceptible Tuberculosis. N Engl J Med.

[3] Dorman SE, Nahid P, Kurbatova EV, Phillips PPJ, Bryant K, Dooley KE, Engle M, Goldberg SV, Phan HTT, Hakim J, Johnson JL, Lourens M, Martinson NA, Muzanyi G, Narunsky K, Nerette S, Nguyen NV, Pham TH, Pierre S, Purfield AE, Samaneka W, Savic RM, Sanne I, Scott NA, Shenje J, Sizemore E, Vernon A, Waja Z, Weiner M, Swindells S, Chaisson RE, AIDS Clinical Trials Group, Tuberculosis Trials Consortium (2021). Four-Month Rifapentine Regimens with or without Moxifloxacin for Tuberculosis. N Engl J Med.

[4] Diriba G, Alemu A, Yenew B, Tola HH, Gamtesa DF, Mollalign H, Eshetu K, Moga S, Abdella S, Tollera G, Kebede A, Dangisso MH (2023). Epidemiology of extensively drug-resistant tuberculosis among patients with multidrug-resistant tuberculosis: A systematic review and meta-analysis. Int J Infect Dis.

[5] Molla KA, Reta MA, Ayene YY (2022). Prevalence of multidrug-resistant tuberculosis in East Africa: A systematic review and meta-analysis. PLoS One.

[6] Wu Y, Zhang Y, Wang Y, Wei J, Wang W, Duan W, Tian Y, Ren M, Li Z, Wang W, Zhang T, Wu H, Huang X (2022). Bedaquiline and Linezolid improve anti-TB treatment outcome in drug-resistant TB patients with HIV: A systematic review and meta-analysis. Pharmacol Res.

[7] World Health Organization (2022). WHO Consolidated Guidelines on Tuberculosis. Module 4: Treatment - Drug-Resistant Tuberculosis Treatment, 2022 Update.

[8] Geiger K, Patil A, Bergman A, Budhathoki C, Heidari O, Lowensen K, Mthimkhulu N, McNabb KC, Mmed NN, Ngozo J, Reynolds N, Farley JE (2024). Exploring HIV disease indicators at MDR-TB treatment initiation in South Africa. Int J Tuberc Lung Dis.

[9] Yuengling KA, Padayatchi N, Wolf A, Mathema B, Brown T, Horsburgh CR, O’Donnell MR (2018). Effect of Antiretroviral Therapy on Treatment Outcomes in a Prospective Study of Extensively Drug-Resistant Tuberculosis (XDR-TB) HIV Coinfection Treatment in KwaZulu-Natal, South Africa. J Acquir Immune Defic Syndr.

[10] Isaakidis P, Cox HS, Varghese B, Montaldo C, Da Silva E, Mansoor H, Ladomirska J, Sotgiu G, Migliori GB, Pontali E, Saranchuk P, Rodrigues C, Reid T (2011). Ambulatory multi-drug resistant tuberculosis treatment outcomes in a cohort of HIV-infected patients in a slum setting in Mumbai, India. PLoS One.

[11] Alemu A, Bitew ZW, Diriba G, Gumi B (2022). Risk factors associated with drug-resistant tuberculosis in Ethiopia: A systematic review and meta-analysis. Transbound Emerg Dis.

[12] Rehman AU, Khattak M, Mushtaq U, Latif M, Ahmad I, Rasool MF, Shakeel S, Hayat K, Hussain R, Alhazmi GA, Alshomrani AO, Alalawi MI, Alghamdi S, Imam MT, Almarzoky Abuhussain SS, Khayyat SM, Haseeb A (2023). The impact of diabetes mellitus on the emergence of multi-drug resistant tuberculosis and treatment failure in TB-diabetes comorbid patients: a systematic review and meta-analysis. Front Public Health.

[13] Sahakyan S, Petrosyan V, Abrahamyan L (2020). Diabetes mellitus and treatment outcomes of pulmonary tuberculosis: a cohort study. Int J Public Health.

[14] Lee EH, Lee JM, Kang YA, Leem AY, Kim EY, Jung JY, Park MS, Kim YS, Kim SK, Chang J, Kim SY (2017). Prevalence and Impact of Diabetes Mellitus Among Patients with Active Pulmonary Tuberculosis in South Korea. Lung.

[15] Siddiqui AN, Khayyam KU, Sharma M (2016). Effect of Diabetes Mellitus on Tuberculosis Treatment Outcome and Adverse Reactions in Patients Receiving Directly Observed Treatment Strategy in India: A Prospective Study. Biomed Res Int.

[16] Lai CC, Tan CK, Huang YT, Chou CH, Hung CC, Yang PC, Luh KT, Hsueh PR (2008). Extensively drug-resistant Mycobacterium tuberculosis during a trend of decreasing drug resistance from 2000 through 2006 at a Medical Center in Taiwan. Clin Infect Dis.

[17] Vilariça AS, Gomes C, Pina J (2008). Análise comparativa entre tuberculose multirresistente e tuberculose extensivamente resistente - Epidemiologia e factores preditivos. Rev Port Pneumol.

[18] Flor de Lima B, Tavares M (2014). Risk factors for extensively drug-resistant tuberculosis: a review. Clin Respir J.

[19] Ngula M, Mildred Z, Mathias T, Ray H, Musso M, Sydney M (2021). Drug-resistant tuberculosis in the northern region of Zambia: A retrospective study. Front Trop Dis.

[20] Kliiman K, Altraja A (2009). Predictors of poor treatment outcome in multi- and extensively drug-resistant pulmonary TB. Eur Respir J.

[21] Frank M, Adamashvili N, Lomtadze N, Kokhreidze E, Avaliani Z, Kempker RR (2019). Long-term follow-up reveals high posttreatment mortality rate among patients with extensively drug-resistant tuberculosis in the country of Georgia. Open Forum Infect Dis.

[22] Kuksa L, Riekstina V, Leimane V, Ozere I, Skenders G, Van den Bergh R, Kremer K, Acosta CD, Harries AD (2014). Multi- and extensively drug-resistant tuberculosis in Latvia: trends, characteristics and treatment outcomes. Public Health Action.

[23] Hatami H, Sotgiu G, Bostanghadiri N, Abadi SSD, Mesgarpour B, Goudarzi H, Migliori GB, Nasiri MJ (2022). Bedaquiline-containing regimens and multidrug-resistant tuberculosis: a systematic review and meta-analysis. J Bras Pneumol.

[24] Lan Z, Ahmad N, Baghaei P, Barkane L, Benedetti A, Brode SK, Brust JCM, Campbell JR, Chang VWL, Falzon D, Guglielmetti L, Isaakidis P, Kempker RR, Kipiani M, Kuksa L, Lange C, Laniado-Laborín R, Nahid P, Rodrigues D, Singla R, Udwadia ZF, Menzies D, Collaborative Group for the Meta-Analysis of Individual Patient Data in MDR-TB treatment 2017 (2020). Drug-associated adverse events in the treatment of multidrug-resistant tuberculosis: an individual patient data meta-analysis. Lancet Respir Med.

[25] Zhuang Z, Sun L, Song X, Zhu H, Li L, Zhou X, Mi K (2023). Trends and challenges of multi-drug resistance in childhood tuberculosis. Front Cell Infect Microbiol.

[26] World Health Organization (2013). Global tuberculosis report 2013.

[27] Tiberi S, Utjesanovic N, Galvin J, Centis R, D'Ambrosio L, van den Boom M, Zumla A, Migliori GB (2022). Drug resistant TB - latest developments in epidemiology, diagnostics and management. Int J Infect Dis.

[28] Biru D, Woldesemayat EM (2020). Determinants of Drug-Resistant Tuberculosis in Southern Ethiopia: A Case-Control Study. Infect Drug Resist.

[29] Naidoo K, Perumal R, Ngema SL, Shunmugam L, Somboro AM (2023). Rapid Diagnosis of Drug-Resistant Tuberculosis-Opportunities and Challenges. Pathogens.

[30] Pedersen OS, Holmgaard FB, Mikkelsen MKD, Lange C, Sotgiu G, Lillebaek T, Andersen AB, Wejse CM, Dahl VN (2023). Global treatment outcomes of extensively drug-resistant tuberculosis in adults: A systematic review and meta-analysis. J Infect.

[31] Singh V, Chibale K (2021). Strategies to Combat Multi-Drug Resistance in Tuberculosis. Acc Chem Res.

[32] Sarathy JP, Gruber G, Dick T (2019). Re-Understanding the Mechanisms of Action of the Anti-Mycobacterial Drug Bedaquiline. Antibiotics (Basel).

[33] Yadav S, Rawal G, Baxi M (2016). Bedaquiline: A Novel Antitubercular Agent for the Treatment of Multidrug-Resistant Tuberculosis. J Clin Diagn Res.

[34] World Health Organization (2022). Rapid communication: key changes to the treatment of drug-resistant tuberculosis.

[35] Mahajan R (2013). Bedaquiline: First FDA-approved tuberculosis drug in 40 years. Int J Appl Basic Med Res.

[36] Sarin R, Vohra V, Singla N, Singla R, Puri MM, Munjal SK, Khalid UK, Myneedu VP, Verma A, Mathuria KK (2019). Early efficacy and safety of Bedaquiline and Delamanid given together in a "Salvage Regimen" for treatment of drug-resistant tuberculosis. Indian J Tuberc.

[37] Borisov SE, Dheda K, Enwerem M, Romero Leyet R, D’Ambrosio L, Centis R, Sotgiu G, Tiberi S, Alffenaar JW, Maryandyshev A, Belilovski E, Ganatra S, Skrahina A, Akkerman O, Aleksa A, Amale R, Artsukevich J, Bruchfeld J, Caminero JA, Carpena Martinez I, Codecasa L, Dalcolmo M, Denholm J, Douglas P, Duarte R, Esmail A, Fadul M, Filippov A, Davies Forsman L, Gaga M, Garcia-Fuertes JA, García-García JM, Gualano G, Jonsson J, Kunst H, Lau JS, Lazaro Mastrapa B, Teran Troya JL, Manga S, Manika K, González Montaner P, Mullerpattan J, Oelofse S, Ortelli M, Palmero DJ, Palmieri F, Papalia A, Papavasileiou A, Payen MC, Pontali E, Robalo Cordeiro C, Saderi L, Sadutshang TD, Sanukevich T, Solodovnikova V, Spanevello A, Topgyal S, Toscanini F, Tramontana AR, Udwadia ZF, Viggiani P, White V, Zumla A, Migliori GB (2017). Effectiveness and safety of bedaquiline-containing regimens in the treatment of MDR- and XDR-TB: a multicentre study. Eur Respir J.

[38] Shaw ES, Stoker NG, Potter JL, Claassen H, Leslie A, Tweed CD, Chiang CY, Conradie F, Esmail H, Lange C, Pinto L, Rucsineanu O, Sloan DJ, Theron G, Tisile P, Voo TC, Warren RM, Lebina L, Lipman M (2024). Bedaquiline: what might the future hold? Lancet Microbe.

[39] Johnson TM, Rivera CG, Lee G, Zeuli JD (2024). Pharmacology of emerging drugs for the treatment of multi-drug resistant tuberculosis. J Clin Tuberc Other Mycobact Dis.

[40] Silva DR, Mello FCQ, Migliori GB (2020). Shortened tuberculosis treatment regimens: what is new? J Bras Pneumol.

[41] Silva DR, Fernandes FF, Ferreira JC, Bernando W, Dalcolmo MMP, Johansen FDC, Mello FCQ (2025). Bedaquiline, pretomanid, linezolid, and moxifloxacin (BPaLM) for multidrug- or rifampin-resistant tuberculosis: a systematic review. J Bras Pneumol.

[42] Nyang'wa BT, Berry C, Kazounis E, Motta I, Parpieva N, Tigay Z, Solodovnikova V, Liverko I, Moodliar R, Dodd M, Ngubane N, Rassool M, McHugh TD, Spigelman M, Moore DAJ, Ritmeijer K, du Cros P, Fielding K, TB-PRACTECAL Study Collaborators (2022). A 24-Week, All-Oral Regimen for Rifampin-Resistant Tuberculosis. N Engl J Med.

[43] James LP, Klaassen F, Sweeney S, Furin J, Franke MF, Yaesoubi R, Chesov D, Ciobanu N, Codreanu A, Crudu V, Cohen T, Menzies NA (2024). Impact and cost-effectiveness of the 6-month BPaLM regimen for rifampicin-resistant tuberculosis in Moldova: A mathematical modeling analysis. PLoS Med.

[44] World Health Organization (2020). WHO consolidated guidelines on tuberculosis: module 4: treatment: drug-resistant tuberculosis treatment.

[45] Günther G, Guglielmetti L, Kherabi Y, Duarte R, Lange C, Tuberculosis Network European Trials group (2024). Availability of drugs and resistance testing for bedaquiline, pretomanid, linezolid, and moxifloxacin (BPaL(M)) regimen for rifampicin-resistant tuberculosis in Europe. Clin Microbiol Infect.

[46] Diacon AH, Dawson R, von Groote-Bidlingmaier F, Symons G, Venter A, Donald PR, van Niekerk C, Everitt D, Winter H, Becker P, Mendel CM, Spigelman MK (2012). 14-day bactericidal activity of PA-824, bedaquiline, pyrazinamide, and moxifloxacin combinations: a randomised trial. Lancet.

[47] Diacon AH, Donald PR, Pym A, Grobusch M, Patientia RF, Mahanyele R, Bantubani N, Narasimooloo R, De Marez T, van Heeswijk R, Lounis N, Meyvisch P, Andries K, McNeeley DF (2012). Randomized pilot trial of eight weeks of bedaquiline (TMC207) treatment for multidrug-resistant tuberculosis: long-term outcome, tolerability, and effect on emergence of drug resistance. Antimicrob Agents Chemother.

[48] Vanino E, Granozzi B, Akkerman OW, Munoz-Torrico M, Palmieri F, Seaworth B, Tiberi S, Tadolini M (2023). Update of drug-resistant tuberculosis treatment guidelines: A turning point. Int J Infect Dis.

[49] Briasoulis A, Agarwal V, Pierce WJ (2011). QT prolongation and torsade de pointes induced by fluoroquinolones: infrequent side effects from commonly used medications. Cardiology.

[50] Wang MG, Liu XM, Wu SQ, He JQ (2023). Impacts of clofazimine on the treatment outcomes of drug-resistant tuberculosis. Microbes Infect.

[51] Xu HB, Jiang RH, Xiao HP (2012). Clofazimine in the treatment of multidrug-resistant tuberculosis. Clin Microbiol Infect.

[52] Du Y, Qiu C, Chen X, Wang J, Jing W, Pan H, Chen W, Liu Y, Li C, Xi X, Yin H, Zeng J, Zhang X, Xu T, Wang Q, Guo R, Wang J, Pang Y, Chu N (2020). Treatment Outcome of a Shorter Regimen Containing Clofazimine for Multidrug-resistant Tuberculosis: A Randomized Control Trial in China. Clin Infect Dis.

[53] Xu C, Pang Y, Li R, Ruan Y, Wang L, Chen M, Zhang H (2018). Clinical outcome of multidrug-resistant tuberculosis patients receiving standardized second-line treatment regimen in China. J Infect.

[54] Duan H, Chen X, Li Z, Pang Y, Jing W, Liu P, Wu T, Cai C, Shi J, Qin Z, Yin H, Qiu C, Li C, Xia Y, Chen W, Ye Z, Li Z, Chen G, Wang S, Liu Y, Chu L, Zhu M, Xu T, Wang Q, Wang J, Du Y, Wang J, Chu N, Xu S (2019). Clofazimine improves clinical outcomes in multidrug-resistant tuberculosis: a randomized controlled trial. Clin Microbiol Infect.

[55] Xu HB, Jiang RH, Xiao HP (2012). Clofazimine in the treatment of multidrug-resistant tuberculosis. Clin Microbiol Infect.

[56] Tang S, Yao L, Hao X, Liu Y, Zeng L, Liu G, Li M, Li F, Wu M, Zhu Y, Sun H, Gu J, Wang X, Zhang Z (2015). Clofazimine for the treatment of multidrug-resistant tuberculosis: prospective, multicenter, randomized controlled study in China. Clin Infect Dis.

[57] Dalcolmo M, Gayoso R, Sotgiu G, D'Ambrosio L, Rocha JL, Borga L, Fandinho F, Braga JU, Galesi VM, Barreira D, Sanchez DA, Dockhorn F, Centis R, Caminero JA, Migliori GB (2017). Effectiveness and safety of clofazimine in multidrug-resistant tuberculosis: a nationwide report from Brazil. Eur Respir J.

[58] Sweeney S, Berry C, Kazounis E, Motta I, Vassall A, Dodd M, Fielding K, Nyang'wa BT (2022). Cost-effectiveness of short, oral treatment regimens for rifampicin resistant tuberculosis. PLOS Glob Public Health.

[59] Chen RH, Burke A, Cho JG, Alffenaar JW, Davies Forsman L (2024). New Oxazolidinones for Tuberculosis: Are Novel Treatments on the Horizon?. Pharmaceutics.

[60] Soriano A, Miró O, Mensa J (2005). Mitochondrial toxicity associated with linezolid. N Engl J Med.

[61] Sotgiu G, Centis R, D'Ambrosio L, Alffenaar JW, Anger HA, Caminero JA, Castiglia P, De Lorenzo S, Ferrara G, Koh WJ, Schecter GF, Shim TS, Singla R, Skrahina A, Spanevello A, Udwadia ZF, Villar M, Zampogna E, Zellweger JP, Zumla A, Migliori GB (2012). Efficacy, safety and tolerability of linezolid containing regimens in treating MDR-TB and XDR-TB: systematic review and meta-analysis. Eur Respir J.

[62] Shaw KJ, Poppe S, Schaadt R, Brown-Driver V, Finn J, Pillar CM, Shinabarger D, Zurenko G (2008). In vitro activity of TR-700, the antibacterial moiety of the prodrug TR-701, against linezolid-resistant strains. Antimicrob Agents Chemother.

[63] Wallis RS, Dawson R, Friedrich SO, Venter A, Paige D, Zhu T, Silvia A, Gobey J, Ellery C, Zhang Y, Eisenach K, Miller P, Diacon AH (2014). Mycobactericidal activity of sutezolid (PNU-100480) in sputum (EBA) and blood (WBA) of patients with pulmonary tuberculosis. PLoS One.

[64] Bruinenberg P, Nedelman J, Yang TJ, Pappas F, Everitt D (2022). Single Ascending-Dose Study To Evaluate the Safety, Tolerability, and Pharmacokinetics of Sutezolid in Healthy Adult Subjects. Antimicrob Agents Chemother.

[65] De Jager V, Gupte N, Nunes S, Barnes GL, van Wijk RC, Mostert J, Dorman SE, Abulfathi AA, Upton CM, Faraj A, Nuermberger EL, Lamichhane G, Svensson EM, Simonsson USH, Diacon AH, Dooley KE (2022). Early Bactericidal Activity of Meropenem plus Clavulanate (with or without Rifampin) for Tuberculosis: The COMRADE Randomized, Phase 2A Clinical Trial. Am J Respir Crit Care Med.

[66] Kumar P, Kaushik A, Lloyd EP, Li SG, Mattoo R, Ammerman NC, Bell DT, Perryman AL, Zandi TA, Ekins S, Ginell SL, Townsend CA, Freundlich JS, Lamichhane G (2017). Non-classical transpeptidases yield insight into new antibacterials. Nat Chem Biol.

[67] Cordillot M, Dubée V, Triboulet S, Dubost L, Marie A, Hugonnet JE, Arthur M, Mainardi JL (2013). In vitro cross-linking of Mycobacterium tuberculosis peptidoglycan by L,D-transpeptidases and inactivation of these enzymes by carbapenems. Antimicrob Agents Chemother.

[68] Hugonnet JE, Tremblay LW, Boshoff HI, Barry CE 3rd, Blanchard JS (2009). Meropenem-clavulanate is effective against extensively drug-resistant Mycobacterium tuberculosis. Science.

[69] Solapure S, Dinesh N, Shandil R, Ramachandran V, Sharma S, Bhattacharjee D, Ganguly S, Reddy J, Ahuja V, Panduga V, Parab M, Vishwas KG, Kumar N, Balganesh M, Balasubramanian V (2013). In vitro and in vivo efficacy of β-lactams against replicating and slowly growing/nonreplicating Mycobacterium tuberculosis. Antimicrob Agents Chemother.

[70] Payen MC, De Wit S, Martin C, Sergysels R, Muylle I, Van Laethem Y, Clumeck N (2012). Clinical use of the meropenem-clavulanate combination for extensively drug-resistant tuberculosis. Int J Tuberc Lung Dis.

[71] Horita Y, Maeda S, Kazumi Y, Doi N (2014). In vitro susceptibility of Mycobacterium tuberculosis isolates to an oral carbapenem alone or in combination with β-lactamase inhibitors. Antimicrob Agents Chemother.

[72] Singh S, Gumbo T, Alffenaar JW, Boorgula GD, Shankar P, Thomas TA, Dheda K, Malinga L, Raj P, Aryal S, Srivastava S (2023). Meropenem-vaborbactam restoration of first-line drug efficacy and comparison of meropenem-vaborbactam-moxifloxacin versus BPaL MDR-TB regimen. Int J Antimicrob Agents.

[73] Gonzalo X, Drobniewski F (2022). Are the Newer Carbapenems of Any Value against Tuberculosis. Antibiotics (Basel).

[74] Nasiri MJ, Zangiabadian M, Arabpour E, Amini S, Khalili F, Centis R, D'Ambrosio L, Denholm JT, Schaaf HS, van den Boom M, Kurhasani X, Dalcolmo MP, Al-Abri S, Chakaya J, Alffenaar JW, Akkerman O, Silva DR, Muňoz-Torrico M, Seaworth B, Pontali E, Saderi L, Tiberi S, Zumla A, Migliori GB, Sotgiu G (2022). Delamanid-containing regimens and multidrug-resistant tuberculosis: A systematic review and meta-analysis. Int J Infect Dis.

[75] Gler MT, Skripconoka V, Sanchez-Garavito E, Xiao H, Cabrera-Rivero JL, Vargas-Vasquez DE, Gao M, Awad M, Park SK, Shim TS, Suh GY, Danilovits M, Ogata H, Kurve A, Chang J, Suzuki K, Tupasi T, Koh WJ, Seaworth B, Geiter LJ, Wells CD (2012). Delamanid for multidrug-resistant pulmonary tuberculosis. N Engl J Med.

[76] Ahmad Khosravi N, Sirous M, Khosravi AD, Saki M (2024). A Narrative Review of Bedaquiline and Delamanid: New Arsenals Against Multidrug-Resistant and Extensively Drug-Resistant Mycobacterium tuberculosis. J Clin Lab Anal.

[77] Ahmed SH, Haider H, Moeed A, Mahmood A, Shivani N, Shuja SH, Hayat J, Jamil B, Fatima R (2024). Efficacy and safety of bedaquiline and delamanid in the treatment of drug-resistant tuberculosis in adults: A systematic review and meta-analysis. Indian J Tuberc.

[78] Marwah V, Patil PR, Choudhary R, Malik V (2023). Early experience of delamanid in extensively drug-resistant pulmonary tuberculosis. Lung India.

[79] Soedarsono S, Mertaniasih NM, Kusmiati T, Permatasari A, Subay S, Adiono SH (2024). Comparison of Individual Regimen Containing Bedaquiline with Delamanid and Bedaquiline without Delamanid on Efficacy and Safety in Multidrug-resistant Tuberculosis Patients: Implementation in Dr. Soetomo General Academic Hospital, Indonesia. Int J Mycobacteriol.

[80] Skripconoka V, Danilovits M, Pehme L, Tomson T, Skenders G, Kummik T, Cirule A, Leimane V, Kurve A, Levina K, Geiter LJ, Manissero D, Wells CD (2013). Delamanid improves outcomes and reduces mortality in multidrug-resistant tuberculosis. Eur Respir J.

[81] von Groote-Bidlingmaier F, Patientia R, Sanchez E, Balanag V Jr, Ticona E, Segura P, Cadena E, Yu C, Cirule A, Lizarbe V, Davidaviciene E, Domente L, Variava E, Caoili J, Danilovits M, Bielskiene V, Staples S, Hittel N, Petersen C, Wells C, Hafkin J, Geiter LJ, Gupta R (2019). Efficacy and safety of delamanid in combination with an optimised background regimen for treatment of multidrug-resistant tuberculosis: a multicentre, randomised, double-blind, placebo-controlled, parallel group phase 3 trial. Lancet Respir Med.

[82] Pontali E, Sotgiu G, Tiberi S, Tadolini M, Visca D, D'Ambrosio L, Centis R, Spanevello A, Migliori GB (2018). Combined treatment of drug-resistant tuberculosis with bedaquiline and delamanid: a systematic review. Eur Respir J.

[83] Vambe D, Kay AW, Furin J, Howard AA, Dlamini T, Dlamini N, Shabangu A, Hassen F, Masuku S, Maha O, Wawa C, Mafukidze A, Altaye K, Sikhondze W, Gwitima T, Keus K, Simelane T, Kerschberger B (2020). Bedaquiline and delamanid result in low rates of unfavourable outcomes among TB patients in Eswatini. Int J Tuberc Lung Dis.

[84] Mok J, Kang H, Hwang SH, Park JS, Kang B, Lee T, Koh WJ, Yim JJ, Jeon D (2018). Interim outcomes of delamanid for the treatment of MDR- and XDR-TB in South Korea. J Antimicrob Chemother.

[85] Das M, Mamnoon F, Mansoor H, Meneguim AC, Singh P, Shah I, Ravi S, Kalon S, Hossain FN, Ferlazzo G, Isaakidis P, Furin J, Acharya S, Thakur HP (2020). New TB drugs for the treatment of children and adolescents with rifampicin-resistant TB in Mumbai, India. Int J Tuberc Lung Dis.

[86] Wells CD, Gupta R, Hittel N, Geiter LJ (2015). Long-term mortality assessment of multidrug-resistant tuberculosis patients treated with delamanid. Eur Respir J.

[87] Nguyen TVA, Anthony RM, Cao TTH, Bañuls AL, Nguyen VAT, Vu DH, Nguyen NV, Alffenaar JC (2020). Delamanid Resistance: Update and Clinical Management. Clin Infect Dis.

[88] Nieto Ramirez LM, Quintero Vargas K, Diaz G (2020). Whole Genome Sequencing for the Analysis of Drug Resistant Strains of: A Systematic Review for Bedaquiline and Delamanid. Antibiotics (Basel).

[89] World Health Organization (2018). Technical report on critical concentrations for drug susceptibility testing of medicines used in the treatment of drug-resistant tuberculosis (WHO/CDS/TB/2018.

